# Oligonucleotide library assisted sequence mining reveals promoter sequences with distinct temporal expression dynamics for applications in *Curvibacter* sp. AEP1-3

**DOI:** 10.1093/synbio/ysaf001

**Published:** 2025-05-21

**Authors:** Maurice Mager, Lukas Becker, Nina Schulten, Sebastian Fraune, Ilka M Axmann

**Affiliations:** Department of Biology, Institute for Synthetic Microbiology, Heinrich Heine University Düsseldorf, Düsseldorf 40225, Germany; Department of Biology, Institute for Synthetic Microbiology, Heinrich Heine University Düsseldorf, Düsseldorf 40225, Germany; Department of Biology, Institute for Zoology and Organismic Interactions, Heinrich Heine University Düsseldorf, Düsseldorf 40225, Germany; Department of Biology, Institute for Synthetic Microbiology, Heinrich Heine University Düsseldorf, Düsseldorf 40225, Germany; Department of Biology, Institute for Zoology and Organismic Interactions, Heinrich Heine University Düsseldorf, Düsseldorf 40225, Germany; Department of Biology, Institute for Synthetic Microbiology, Heinrich Heine University Düsseldorf, Düsseldorf 40225, Germany

**Keywords:** *Curvibacter* sp. AEP1-3, *Hydra vulgaris* AEP, promoter, FACS, genome mining

## Abstract

The *β-proteobacterial* species *Curvibacter* sp. AEP1-3 is a model organism for the study of symbiotic interactions as it is the most abundant colonizer of *Hydra vulgaris*. Yet, genetic tools for *Curvibacter* are still in their infancy; few promoters have been characterized so far. Here, we employ an oligonucleotide-based strategy to develop novel expression systems *Curvibacter*. Potential promoters were systematically mined from the genome *in silico*. The sequences were cloned as a mixed library into a mCherry reporter vector and positive candidates were selected by Flow Cytometry to be further analysed through plate reader measurements. From 500 candidate sequences, 25 were identified as active promoters of varying expression strength levels. Plate reader measurements revealed unique activity profiles for these sequences across growth phases. The expression levels of these promoters ranged over two orders of magnitudes and showed distinct temporal expression dynamics over the growth phases: while three sequences showed higher expression levels in the exponential phase, we found 12 sequences saturating expression during stationary phase and 10 that showed little discrimination between growth phases. From our library, promoters of the genes *dnaK, rpsL* and an acyl-homoserine-lactone (AHL) synthase stood out as the most interesting candidates fit for a variety of applications. We identified enriched transcription factor binding motifs among the sorted 33 sequences and genes encoding for homologs of these transcription factors in close proximity to the identified motifs. In this work, we show the value of employing comprehensive high-throughput strategies to establish expression systems for novel model organisms.

## 1 Introduction

*Curvibacter* sp. AEP1-3 (hereafter *Curvibacter*) is a rod-shaped *β-proteobacterial* species best known for its symbiotic interaction with *Hydra vulgaris* (hereafter *Hydra*) [1], a freshwater polyp of the basal metazoan phylum Cnidaria, a sister group to the Bilateria. Together with other members of *Hydras* microbiota, they form a complex system of bacteria-bacteria as well as bacteria–host interactions -[2–4]. While the host provides an ecological niche to its colonizers, the microbiota affects mobility, asexual reproduction as well as protection against a fungus of the genus *Fusarium* [1]. This symbiotic relationship provides an invaluable avenue for the study of interkingdom interactions and allows for the exploration of general principles of symbiosis in the natural world such as the remarkable host-microbe communication between *Curvibacter* and *Hydra* established through the exchange of N-acetyl homoserine lactone [2].

The limited genetic accessibility of *Curvibacter* restricts advancements in the genetic manipulation of its cells, thereby impeding progress in the field of interkingdom symbiosis. Genomic modifications over homologous recombination [5] are cumbersome [6] and have a low success rate. *Curvibacter* cells are amenable to transformation using RSF1010 vector [7] constructs through conjugation with *E. coli* donor cells but only a limited number of promoters are accessible for use and none of them have been characterized to date. In this study, we set out to develop a strategy to create a tool kit of novel promoters for *Curvibacter* to promote its use as a model organism.

The Anderson Collection of Synthetic Promoters is a good reference for the development of orthogonal constitutive expression systems (http://parts.igem.org/Promoters/Catalog/Anderson). The collection provides a range of expression levels that have been well characterized in many species such as *E.coli*, *V. natriegens* and some *Cyanobacteria* [8  9] but as orthogonal promoters they usually show the same temporal expression dynamics in the form of a stable, constant activity over growth conditions, providing the same transcript level over the entire time of cultivation. However, when expressed from a plasmid, the total expression level of most promoters often increases significantly in the stationary phase due to changes in the copy number of most plasmids: the copy number generally increases with slower growth during the stationary phase, leading to higher transcript expression and increased protein levels [10–12]. While this may be either desirable for some applications or irrelevant in experimental setups where cells are only observed during logarithmic growth, such accumulation may prove detrimental, for example, in long-term experiments. Therefore, for the development of plasmid-based expression systems with stable expression in different growth phases, orthogonal promoters that cannot absorb this burst of expression may not be the most suitable choice.

While hand-picking or designing individual sequences with a predicted expression strength is a valid strategy for developing expression systems, the use of entire sequence libraries provides a promising alternative due to the high throughput of tested sequences [13]. Such libraries are generated by synthesizing oligonucleotide sequences on highly sophisticated commercial DNA synthesis platforms that allow the simultaneous generation of many sequences at the same time. These libraries are collected and purified in a single sample and can be used for cloning applications to create a library of plasmids each containing a different synthesized sequence, as well as, in our case, a reporter sequence such as the red fluorescence protein mCherry or the Green Fluorescence Protein (GFP). Downstream, the use of flow cytometry and cell sorting can aid in picking positive candidates from such libraries to avoid the extensive effort of manually picking and analyzing individual colonies. The aforementioned libraries can consist of sequences with varying degrees of randomization, generated by using mixed nucleotides during synthesis which allows for the incorporation of any base by chance [14]. This strategy is necessary for projects in which the investigators aim to obtain the best-suited sequences from a bias-free sequence space or if there is no information available that could reduce the degree of freedom. The GeneEE library of Lale *et al.* [15] follows exactly this approach by using long stretches of randomized nucleotides to find novel promoter sequences *de novo*.

An alternative strategy we employ here is the generation of highly curated sequence libraries. Limiting the pool only to sequences with a high probability of success simplifies downstream processes and can yield many more positive candidates in significantly smaller libraries, making it an easier and more cost-efficient method. The generation of such libraries can be facilitated by neural networks, trained on existing promoter sequences, extracted from curated libraries such as the Prokaryotic Promoter Database (PDD) [16] or as in our case simply by using existing sequences harvested directly from the target species genome [17]. The latter approach results in finding promoter sequences that would not be orthogonal, but it is a valid approach to also find expression systems, which are either inducible or show a desired temporal expression dynamic in certain growth phases. Moreover, these sequences already inherit the genomic context for specific regulation, to a degree. By extracting those sequences, it allows the identification of different regulatory elements depending on the extracted length and culture conditions.

**Figure 1.**
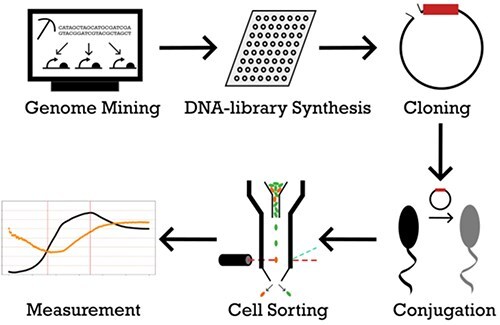
Schematic overview of the applied workflow.

The aim of this study is the discovery and characterization of novel expression systems for the use in expression vectors for *Curvibacter*, with a special focus on promoters that provide stable expression independent of growth phases in liquid media. To increase the odds of individual promoter sequences, promoter, and ribosomal sequences were not further discriminated, but entire 5ʹ untranslated regions (5ʹ UTRs) were used instead. Candidate sequences for this study were directly harvested (Figure 1) from the *Curvibacter* genome sequence (GCF_002163715.1). Sequences were ordered as single-stranded oligonucleotides and cloned into an expression vector with mCherry as a reporter gene. mCherry expression serves in this work as a quantifiable proxy for the expression strength of a given candidate sequence. Positive candidates were selected with the aid of flow cytometry and cell sorting and were subsequently analysed via bulk fluorescence measurement. The extracted 5ʹ UTRs displayed a wide range of activity levels that can be used for different applications. We discovered different behaviour of the selected candidates in terms of their activity over growth phases: while most candidate 5ʹ UTRs showed typically increased activity in the late exponential to stationary phase, we also found some 5ʹ UTRs to be slightly more active in exponential than in stationary phase. Several sequences were found to show similar expression levels during the exponential as well as stationary phases, highlighting how useful oligonucleotide library based approaches are in finding promoter sequences with desired features.

Design of the oligonucleotide library starts by mining the host genome for suitable promoter sequences. Sequences are synthesized, cloned, and imported into the host species and sorted for activity by Flow Cytometry Cell Sorting. In-depth assays of individual sequences characterize each candidate promoter in detail.

## 2 Material and methods

### 2.1 Extraction of promoter sequences in the genome of *Curvibacter* sp. AEP1-3

To extract candidate promoter sequences suitable for our experiments, first, we retrieved the GenBank file for the *Curvibacter* genome from the GenBank FTP site, serving as our primary source of genomic data. All subsequent data processing and analysis were performed in the Python 3 programming environment within the Jupyter Notebook development platform. To extract and manage genomic data, we utilized the BioPython library. The scripts and all relevant data files can be found on our GitHub repository (https://github.com/Kanomble/curvibacter_promotor_studies).

Local start and stop positions as well as the genomic orientation of all sequence elements labelled as ‘genes’ within the *Curvibacter* genome were structured into a dictionary. Gene sequence identifiers were employed as keys but simultaneously also stored in a list object, preserving their exact positions within the genome. This list was instrumental in tracking sequence location and order. To provide context for the gene sequences, we implemented a parsing process to retrieve information about the current gene sequence, the previous gene sequence, and the next gene sequence based on the current identifier. To refine our dataset, we filtered out sequences that did not meet length criteria, specifically sequences with more than 170 base pairs and less than 60 base pairs, ensuring that only appropriately sized 5ʹ UTRs were included in the analysis. Further filtering steps involve removing sequences with opposing orientations and overlapping segments. To remove potential tRNA and rRNA 5ʹ UTRs or 5ʹ UTRs without any activity the resulting data frame was merged with a data frame of a previously conducted transcriptome analysis of an RNAseq experiment with *Curvibacter* wildtype cells (Further details s. Pietschke *et al.* 2017 [2]). The merging step eliminated all potential promoter sequences from genes that are not part of the transcriptome analysis, and as a result, these genes do not exhibit any expression levels in the standard R2A growth media of *Curvibacter*. In addition, it excluded all genes labelled as tRNA or rRNA since they were not present in the transcriptome data frame. This step was vital in eliminating conflicting or redundant information within the analysed gene sequences. In the next step binding sites for the restriction enzymes BsaI, BsmBI, and BbsI were identified among the sequences. Sequences containing these binding sites were excluded. After this 722 candidates remained in the data frame. As the sequence synthesis order was capped to 500 sequences this selection was cut further: the 350 smallest sequences with all sequences from 60 to 98 bp were included first, as the sequence synthesis order was capped to a maximum length of 150 bp including added restriction sites on each site. The remaining sequences were sorted by their read counts derived from the RNAseq experiment mentioned earlier. As the read counts encompassed three orders of magnitudes, the 50 5ʹ upstream regions from genes with reads of each order of magnitude were picked to cover a wide range of potential expression levels. All sequences were subsequently trimmed from the 5ʹ end to reach a length of 98 base pairs. Restriction sites for golden gate cloning were then added, followed by the insertion of random bases behind the restriction site to meet the synthesis specifications, ensuring that all sequences reached a uniform length of exactly 150 base pairs.

### 2.2 Transcriptome read mapping

Raw RNAseq reads were obtained as described in Pietschke *et al.* 2017 [2]. Briefly, RNA was isolated with the RNeasy Mini Kit (Qiagen) from *Curvibacter* grown in R2A media at $18^{\circ}\mathrm{C}$ to mid-exponential growth phase. cDNA libraries were constructed using the TrueSeq Stranded mRNA LT-RiboZero Kit (Illumina) and cDNA libraries were sequenced using a NextSeq 500 machine (Illumina) in paired-ends mode. Before mapping the obtained sequences against the reference genome of *Curvibacter*, the sequences were subjected to quality control and preprocessing. Sequences were trimmed using trimmomatic (Version 0.39, [18]). Trimmed FASTQ files were analysed for quality using FASTQC (Version 0.11, [19]). Subsequently, the preprocessed sequences were mapped against the reference genome of *Curvibacter* utilizing the Kallisto mapper (Version 0.50, [20]). RAW sequences are uploaded to NCBI as BioProject PRJNA1082616. The relevant trimming and mapping procedure can be found in this GitHub repository: (https://github.com/Kanomble/curvibacter_transcriptomics). In this work, we used the results of the RNA-Seq analysis only to filter the extracted 5ʹ UTRs (see earlier section).

### 2.3 Library golden gate cloning

In total, 40 ng/kb of DNA library (but a minimum of 5 ng per reaction) were added to 20 ng/kb of entry vector (Genbank file available on GitHub https://github.com/Kanomble/curvibacter_promotor_studies) to maximize the yield of successful integration without compromising efficiency. Golden Gate Reaction was performed according to the LVL2 Golden Gate assembly protocol as described by Marillonet *et al.* [21], but the final digest duration was increased to 1 h to reduce entry vector religation. In total, 50 µl of highly competent Dh5 Alpha cells were transformed with 10 µl of Golden Gate reaction and plated on four selection plates to reduce colony crowding. After 24 h of growth, a minimum of 5.000 (for a library with 500 sequences) colonies of equal size were obtained and scraped off the plates. Plasmid DNA was extracted from the cell mixture using a standard MiniPrep kit from Macherey Nagel. The *E. coli* donor strain for conjugation into *Curvibacter* was retransformed with the plasmid library to yield a minimum of 5.000 colonies.

### 2.4 Conjugation of library vectors into *Curvibacter* sp. AEP1-3 glmS::GFP

*Curvibacter* with a GFP insertion in the *glmS* locus was obtained from Nawroth *et al.* [22]. The GFP carrying *Curvibacter* was inoculated from a fresh plate into R2A+ media and grown for 36h to stationary phase prior to conjugation. DAP auxotroph *E. coli* donor cells were directly scraped off transformation plates, washed in LB media, and used for conjugation.

Three ml of *E. coli* donor cells at OD1 and 5 ml of *Curvibacter* stationary phase at OD2 were mixed and the conjugation mix was centrifuged at 5000 rpm for 5 minutes. Cells were washed in one ml of R2A+ and centrifuged as before. Cells were resuspended in 100 µl of R2A+ and spotted on four plates of R2A media without the addition of diaminopimelic acid (DAP) or antibiotics.

Plates were incubated overnight at $30^{\circ}\mathrm{C}$ and cell spots were scraped off and washed in one ml of R2A+. Then, 50 µl of the conjugation mixture was separately plated on an R2A plate containing the respective antibiotic for quality control. The rest of the mixture was centrifuged, resuspended in 200 µl of the remaining media, and spread equally over eight R2A plates containing the respective antibiotic. If the colony count on the quality control plate was higher than 250, the total conjugation yielded over 5000 conjugation events and the plates could be used further. Conjugation plates were scraped and *Curvibacter* cells were washed in one ml of R2A+. Cells were diluted to OD 0.02 in R2A and sorted as described later.

### 2.5 Flow cytometry and cell sorting

*Curvibacter* cells were sorted using the CytoFlex SRT Benchtop Cell Sorter. Forward scatter (FSC) and side scatter (SSC) were measured using a 488 nm laser and a 488/8 nm Bandpass filter. Violet side scatter (VSSC) was measured using a 405 nm laser and a 405/5 nm Bandpass filter. GFP fluorescence was measured using a 488 nm laser and a 525/40 nm Bandpass filter. mCherry fluorescence was measured using a 561 nm laser and a 610/20 nm Bandpass filter. The following gain settings were used, see Table 1:

**Table 1.** Gain settings for flow cytometry

Filter	Gain setting (X/3000)
FSC	76
SSC	299
VSSC	106
GFP	196
mCherry	1216

The cell population of interest was sorted based on a mCherry fluorescence higher than the background signal. To determine the gate for background fluorescence, the background strain *Curvibacter* sp. AEP1-3 *glmS*::GFP was used and the gate was set to exclude 99% of this population. To normalize the variation of fluorophore signal strength variation based on factors such as cell size, polar aggregation of fluorophores, and/or cell cycles [23], GFP signal strength was used to contextualize RFP signal strength as follows: the subpopulation was split into four quadrants based on their GFP and mCherry signal: green fluorescence from the genomic GFP from 100.000 to 500.000 AU was considered ‘high green’, from 25.000 to 70.000 was considered ‘low green’ fluorescence. Equally, red fluorescence from 8.000 to 90.000 AU was considered ‘high red’, and from 3.500 to 4.000 was considered ‘low red’ fluorescence. The quadrant of each sorted cell is stated in the inventory list (see the positive candidate table on GitHub). In total, 2000 Cells were sorted into four tubes containing 100 µl of R2A based on their combined red and green fluorescence signal.

This mixture was plated on R2A plates and incubated at $30^{\circ}\mathrm{C}$ for 48 h. Colonies of surviving cells were tested for successful promoter integration in the expression vector using cPCR and Sanger Sequencing. Colonies were inoculated in one ml of R2A+ containing the respective antibiotic and grown for 48 h. One ml of 50% (v/v) glycerol in distilled water was added and cultures were frozen at $-80^{\circ}\mathrm{C}$ for further use.

### 2.6 Bulk fluorescence intensity measurements

*Curvibacter* cells containing one of the selected 5ʹ UTRs were inoculated from a fresh R2A+ plate into R2A+ media and grown for 36 h at $30^{\circ}\mathrm{C}$ in 24 well plates in a BMG labtech Clariostar plate reader until stationary phase. From these precultures, the main cultures were inoculated to an OD of 0.05. Growth and fluorescence were monitored over 36 h. mCherry fluorescence from the reporter constructed was monitored at 570/15 nm bandwidth excitation and 620/20 nm bandwidth emission and GFP fluorescence from the genomically integrated GFP was monitored at 470/15 nm bandwidth excitation and 515/20 nm bandwidth emission. The measurements were performed in individual repetitions of three for each candidate of the *Curvibacter* mutants.

### 2.7 *Curvibacter* growth media

R2A+ media was prepared by adding additional nutrients to premixed R2A from Carl Roth.

**Table 2.** R2A+ media composition.

Ingredient	Amount (per 1L)
R2A (premixed	3 g
Peptone	4 g
Glucose	2.5 g
Yeast extract	1 g
Distilled water	up to 1L

### 2.8 Mathematical operations for RFU assessment

Fluorescence intensity measurements were adjusted to eliminate background signals by employing a media-only control well. The corrected fluorescence intensity for a specific fluorophore was obtained by subtracting the signal in the presence of media control from the signal of that fluorophore alone.


(1) $$ FI(fluorophore) = signal(fluorophore) - signal(media\ control) $$

The fluorescence intensity FI(fluorophore) is the value for the emission intensity of the measured fluorophore [mCherry or GFP, signal(fluorophore)] subtracted by the background emission of the media control [R2A+, signal(media control)].

To account for variations in biomass, relative fluorescence units (RFUs) were further normalized using the GFP intensity as a reliable proxy for biomass. This normalization was carried out as follows:


(2) $$ RFU = FI(mCherry) / FI(GFP) $$

In order to assess changes in 5ʹ UTR activity across different growth phases, FI values were compared between the mid-exponential and stationary phases. This calculation was performed using the formula:


(3) $$\begin{aligned}& Differential\ activity \nonumber\\& \quad = \frac{Normalized\ FI\ values(mid-exponential\ growth\ phase)}{Normalized\ FI\ values(stationary\ growth\ phase)} \end{aligned}$$

Values below 0.7 indicated 5ʹ UTRs more active during the stationary phase, values above 1.3 suggested greater activity in the exponential phase, and values falling in between were indicative of 5ʹ UTRs that did not show a significant preference for either growth phase. This method allowed for a comprehensive assessment of 5ʹ UTR behaviour in relation to different growth phases.

The datasets obtained from the plate reader (BMG labtech clariostar), which included Biomass and FI data points for mCherry and GFP, underwent a filtering process to remove outliers and reduce noise. This smoothing step utilized the savgol_filter function from the Python scipy package, implementing the Savitzky–Golay smoothing technique [24]. The Savitzky–Golay smoothing filter is a data processing method commonly employed in signal processing and data analysis. Its purpose is to smooth noisy data while preserving essential signal features. This step was taken to enhance the accuracy of determinations regarding stationary and exponential growth phases.

The time points for defining the mid-exponential and stationary phases were determined by applying the Savitzky–Golay smoothing technique to the raw OD600 values and calculating the maximum slope for the mid-exponential phase, as well as the maximum OD600 value for the stationary phase.

### 2.9 Inference of transcription factor binding motifs

To identify potential transcription factor binding sites and motifs (TF-Motifs), the nucleotide sequences of the 33 5ʹ UTRs identified via Flow Cell Cytometry were used as input sequences for the XSTREME algorithm ([25]). The XSTREME algorithm conducts a comprehensive motif analysis. It was configured with default settings to search for binding motifs within the prokaryotic CollectTF database (http://www.collectf.org/browse/home/), which contains known bacterial transcription factor binding sites. The proteins corresponding to the TF motifs were downloaded from UniProt and combined into a FASTA file. Using this combined FASTA file as a query, a BLAST (Basic Local Alignment Search Tool; [26; 27]) search against the *Curvibacter* proteome was conducted with the standalone BLAST tool from NCBI ([28]). The parameters were changed as follows: e_value=0.001, num_alignments=10 000, and word_size=3.

## 3 Results

### 3.1 Development of a streamlined workflow for 5ʹ UTR mining

We designed a synthetic biology approach for 5ʹ UTR mining in sequenced bacterial species to develop novel expression systems. The details of the workflow to filter transcriptionally active 5ʹ UTRs are described in the first methods section in detail. Briefly, candidate 5ʹ UTRs for this study were directly harvested from the *Curvibacter* genome sequence. As the *Curvibacter* genome contains 4096 predicted genes, nearly the same amount of intergenic regions (as some genes overlap) exist as potential regulatory sites. Filtering steps removed intergenic regions of divergent genes; with a length < 60 and > 170bp; 5ʹ UTRs of tRNA and rRNA genes as well as those intergenic regions containing restriction sites of BsaI, BsmBI, and BbsI. A table of all ordered sequences is available as a candidate table together with the complete workflow on GitHub (https://github.com/Kanomble/curvibacter_promotor_studies) and can be adapted to any bacterial genomic sequence. In the following, we show how representative our selection is for the genome of *Curvibacter* sp. AEP1-3.

### 3.2 Representativeness of extracted 5ʹ UTRs in *Curvibacter* sp. AEP1-3

**Figure 2.**
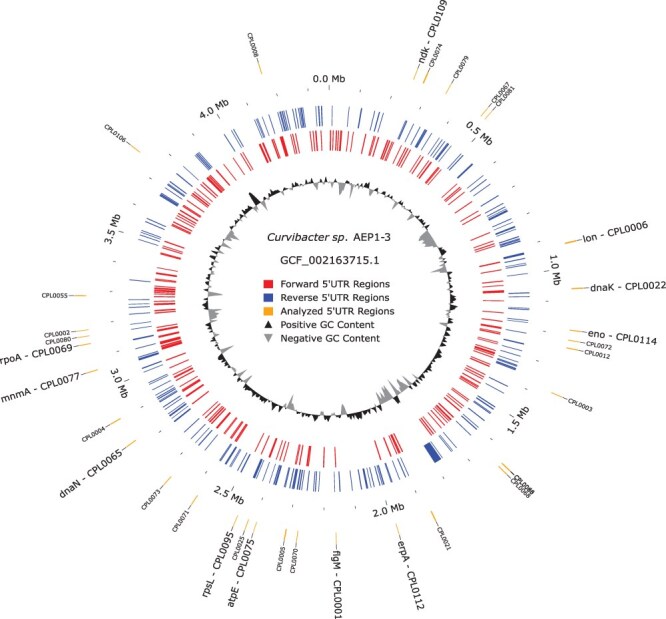
Distribution of extracted 5ʹ UTRs within the genome of *Curvibacter*.

**Figure 3.**
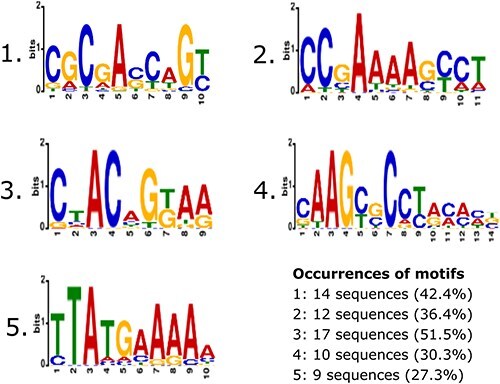
Conserved sequence motifs within the analysed 33 5ʹ UTRs of *Curvibacter*.

The 33 5ʹ UTRs that were confirmed by Flow Cytometry (see next section) are evenly distributed throughout the entire *Curvibacter* genome (see Figure 2). The length of the confirmed 5ʹ UTRs ranges from 60 to 146 bp. Further, sequences of these confirmed candidate sequences were analysed for the occurrence of common motifs (see Figure 3).

The innermost circle is composed of a density plot that showcases the GC content of the respective genome regions. Positive GC-content refers to genomic regions where the GC-content is higher than the genome-wide average, whereas negative GC-content refers to regions where it is lower. The two following red and blue circles highlight the initially extracted 500 5ʹ UTRs. Blue lines correspond to genes with a forward orientation (clockwise), red lines vice versa. The outer circle represents the 33 via Flow Cytometry sorted 5ʹ UTRs. 5ʹ UTRs of CDS regions labelled as ‘hypothetical protein’ or with protein names that are too long are not labelled.

Sequence motifs are part of the CollectTF database. Figure highlights the 5 most prevalent motifs, additional motifs can be found in Supplementary Table S1.

**Figure 4.**
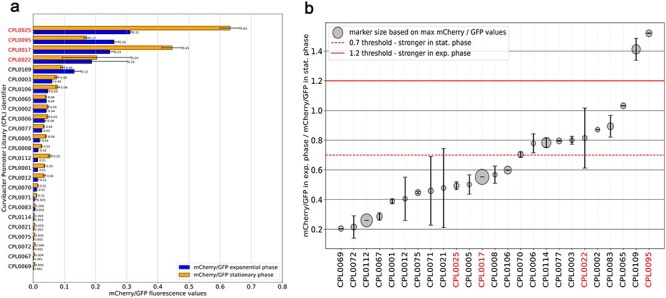
5ʹ UTR expression levels as measured by mCherry/GFP levels.

Among the 33 5ʹ UTRs, five distinct transcription factor binding site motifs (TF-Motifs) have been identified using the XSTREME algorithm from the MEME-suite portal, with a setup that enables searching for TF-Motifs within the CollectTF database for bacterial transcription factor binding sites [25]. The identified motifs are found in several transcriptional regulators. For instance, the first motif (1) exhibits similarities to TF-Motifs of the AmrZ and LasR proteins of *Pseudomonas aeruginosa* [29; 30]. AmrZ serves as a transcriptional activator and/or repressor of virulence factors, as well as genes involved in environmental adaptation. LasR, on the other hand, serves as a transcriptional activator of the elastase structural gene LasB [31] and it is considered as a transcriptional activator for virulence genes in *Pseudomonas aeruginosa* [32]. In 23 of 25 5ʹ UTRs that were further analysed by bulk fluorescence measurement (further referred to as candidate 5ʹ UTRs) (see Figure 4a) at least one of the inferred TF-Motifs has been identified (see positive candidate table on GitHub). A detailed description of all identified motifs can be found there as well.

**A: 5ʹ UTR expression levels as measured by mCherry/GFP levels within the exponential growth phase and stationary growth phase.** 5ʹ UTR sequences are sorted in decreasing order based on the mCherry/GFP value during the exponential growth phase. The red-marked CPL identifiers represent the highlighted sequences in the section later. **B: Relative expression levels of 5ʹ UTRs during exponential phase and stationary phase.** 5ʹ UTRs with values under 0.7 are more active during the stationary growth phase, while 5ʹ UTRs with values above 1.2 are considered to be more active during the exponential growth phase. Dot size correlates with overall expression level. The red-marked CPL identifiers represent the highlighted sequences in the section later. Data points represent the average of three repetitions. Error bars display the standard deviation.

BLAST analysis of the UniProt sequences obtained from the TF motifs revealed 38 unique homologous proteins from five of the eleven UniProt sequences within *Curvibacter* (see BLAST result table on GitHub). The DosR (*Mycobacterium tuberculosis*), LasR (*Pseudomonas aeruginosa*), CcPa (*Streptococcus pneumoniae*), LexA (*Vibrio parahaemolyticus*), and ExpR (*Rhizobium meliloti*) proteins, with 28, 5, 5, 3, and 5 homologous protein hits in *Curvibacter*, respectively. Six *Curvibacter* homologs appear multiple times in the result dataset. Transcription factors, response regulators, autoinducer-binding domain containing, and chemotaxis proteins are among the homologous protein hits. None of the query sequences share a direct homology to the associated proteins of the described 33 5ʹ UTR regions. However, the LasR protein is a homolog to the CurR1 and CurR2 (Refseq protein identifier: WP_087495460.1 and WP_087496729.1) proteins in *Curvibacter*, which are described in Pietschke *et al.* and function directly as response regulators for the acyl-homoserine-lactone (AHL) synthases Curl1 and Curl2 (WP_232460033.1 and WP_232459811.1) in *Curvibacter* [2]. Interestingly, with CPL0025 we identified an 5ʹ UTR that is associated with the AHL synthase Curl2. Among the BLAST results, the response regulator transcription factor sequence WP_157673178.1 was identified as homologous to the query protein LasR, while both WP_157673178.1 and WP_087495595.1 showed homology to the DosR query protein. They are located near CPL0077, which encodes a protein annotated as tRNA synthase MnmA, and CPL0071, which is annotated as a transferrin family substrate-binding protein (WP_087495999.1 and WP_087495596.1). Both, the response regulator WP_087495105.1 and the substrate-binding domain-containing protein WP_087495120.1 of *Curvibacter* are homologous to DosR and CcPa and are located near the 5ʹ UTR of CPL0112 (associated with the ErpA protein WP_087495111.1).

### 3.3 *Curvibacter* strains carrying functional reporter constructs show a range of expression levels

Cells carrying one of 33 unique reporter constructs sorted by flow cytometry were further analysed for their expression level throughout different growth phases (see Figure 4a) using bulk fluorescence measurement in a plate reader (full list of candidates is available on GitHub as candidate table). The candidates vary in length and GC content. For instance, CPL0025 has a length of 76 bp with a GC content of 40%. In contrast, CPL0095 is 113 bp long with a GC content of 42%, and CPL0022 spans 123 bp with a GC content of 46%. From 33 total sorted candidates, 25 showed detectable expression levels and were therefore included in the following analysis.

**Figure 5.**
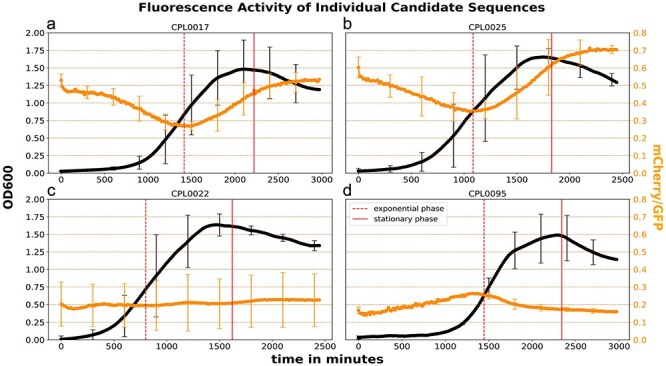
5ʹ UTR expression levels for the highlighted sequences CPL0017 (control), CPL0025, CPL0022, and CPL0095.

All strains are based on the same *Curvibacter* background strain containing a genomic GFP integration [22] in the *glmS* locus with a constant expression level relative to biomass until the early stationary phase (see Supplementary Figure S2). As this GFP signal was less noisy compared to OD measurements at optical densities near OD 0.1 and as the fluorophore accumulation in the late stationary phase due to protein aggregation and the lack of growth phase dependant regulation was nearly identical for both the reporter mCherry construct and the genomic GFP integration, the GFP signal was further used as a normalization factor for the mCherry signal (see equation three in Mathematical Operations for RFU Assessment). This reduces noise in low optical density cultures and leads to a stable signal in the later stationary phase, making it easier to determine reporter activity during the exponential and stationary phases. RFUs are therefore given as the fraction of mCherry/GFP intensity. Supplementary Figures S1-S4 contain a comparison of biomass and raw GFP expression in the background strain containing CPL0022 or CPL0017 in linear or log scale, respectively. Supplementary Figure S2 shows the correlation between GFP and biomass of the background strain, showing that GFP expression remains constant relative to biomass until the stationary phase is reached and linearly increases after reaching the stationary phase in the same way that it does in trans-expression systems, effectively negating this drift. Figure 5 a-d shows a linear relation for mCherry/GFP during the stationary phase as a result of this.

Full and dotted lines represent time point of exponential and stationary phase, respectively, which was used for Figure 4B. RFU (orange line) equals mCherry intensity (candidate 5ʹ UTRs) normalized to GFP intensity (which is located on the genome). Data points represent the average of three repetitions. The standard deviation of the OD and RFU values is displayed as an error bar for every 20th value.

Figure 4a shows the RFUs of mCherry normalized to GFP for all candidates, which showed expression levels above the background noise. The above values serve as a proxy for the relative expression levels of their corresponding 5ʹ UTRs during the exponential phase and the stationary phase and should guide investigators in picking expression systems for their specific use case. Expression levels range from 0.61 to 0.005 relative to GFP in the stationary phase, encompassing two orders of magnitude in terms of expression strength. Among the 25 analysed candidate sequences, 12 show less than 75% of activity during the exponential phase compared to the stationary phase, while 10 display relatively consistent expression strength regardless of the current growth phase (Figure 4b). Additionally, three candidate sequences demonstrate at least 125% activity during the exponential growth phase compared to the stationary phase.

### 3.4 Activity level of candidate 5ʹ UTRs shows distinct temporal expression dynamics over growth phases

In this section we provide detailed information on the measured fluorescence activity over time of three (plus CPL0017 as control) selected candidate 5ʹ UTRs of *Curvibacter*. We recommend these 5ʹ UTRs for further experimental use as they cover a range of different temporal expression patterns and strengths. Additionally, we provide the graphical analysis of the 21 remaining 5’ UTRs in our GitHub repository (https://github.com/Kanomble/curvibacter_promotor_studies).

The CPL0025 (Figure 5b) sequence is the 5ʹ UTR of the gene AEP_RS11205, expressing the AHL synthase (RefSeq protein identifier: WP_232459811), also described as Curl2 in Pietschke *et al.* [2]. The full promoter region (519 bp) of the AHL synthase Curl2 is activated by homoserine lactones, bacterial quorum sensing molecules which play a crucial role in regulating gene expression in response to population density [33]. Here, we show that even the smaller 5ʹ UTR region of 76 bp could drive the expression of our reporter construct. The 5’ UTR shows lower expression levels during exponential growth and elevated expression levels after entering the stationary phase. CPL0025 was the strongest candidate among all tested 5ʹ UTRs, surpassing even the expression level of the highly active J23100-RBS* 5ʹ UTR (CPL0017), which was used as reference (Figure 5a). A TF-Motif similar to the AHL-activated transcriptional regulator LasR (see Supplementary Table S1) was not detected within the 76 bp long 5’ UTR of CPL0025. The only motif found in this 5’ UTR is similar to TF-Motifs found in Gram-positive bacteria, the motif is located within the positions 20–29 (TTACAAGAAA) of the 5’UTR. Specifically, similar to motifs of the global transcriptional regulator CodY from *Lactococcus lactis* and *Streptococcus pyogenes* as well as for CcpA from *Streptococcus pneumoniae* (see Figure 3 5. and Supplementary Table S1) [34–37].

Sequence CPL0022 (Figure 5c) is the 5ʹ UTR of the gene AEP_RS05045 expressing *dnaK* (RefSeq protein identifier: WP_087494375), a molecular chaperone protein of the (Heat-shock-protein 70) Hsp70 family. The *dnaK* candidate 5ʹ UTR shows a very constant expression level throughout all growth phases in *Curvibacter* compared to all other tested 5ʹ UTRs, with minor bursts of transcriptional activity during late exponential and early stationary growth phases. In comparison with other sequences in this study, the *dnaK* shows a very constant expression level throughout all growth phases, a relatively high expression level, and very little bias towards growth phases. CPL0022 contains 9 bp long TF-Motif for LasR binding within the positions 12–21 (CACAACCAGC) of the 5’ UTR sequence. Additionally, CPL0022 contains a CodY motif from *Bacillus anthracis* and an ExpR motif from *S. melliloti* (see Supplementary Table S1).

CPL0095 (Figure 5d) is the 5ʹ UTR of the gene AEP_RS11420 (RefSeq protein identifier: WP_011466063) expressing *rpsL*. RpsL is a 12S protein component of the 30S ribosomal subunit. This candidate 5ʹ UTR displays high activity during the exponential phase, with a steady increase in activity until the mid-exponential phase. The activity then decreases to approximately half of its maximum during the stationary phase. CPL0095 contains a range of sequence motifs, such as a LasR motif from *P. aeruginosa*, CodY from *B. anthracis* as well as *S. pyogenes*, and a LexA motif from *V. parahaemolyticus* (see Supplementary Table S1).

## 4 Discussion

### 4.1 25 novel promoters for the use in *Curvibacter* show distinct temporal expression dynamics

As *Curvibacter* is a promising model organism we set out in this project to extract novel expression systems for this species from a self-designed oligonucleotide library. This library was generated by mining the *Curvibacter* genome for 5ʹ UTR sites in an automated fashion. Positive candidates were first picked via Flow Cytometry and subsequently individual sequences were analysed by bulk fluorescence measurement. From our 500 initial candidate sequences, we found 25 positive candidates that showed expression based on our reporter plasmid. Among these, we could find expression levels over two orders of magnitude and a variety of different temporal expression dynamics over growth phases (see Figure 4a). We found 12 candidate 5ʹ UTRs which show a higher expression level in the stationary phase compared to the exponential phase. In total, 10 candidate 5ʹ UTRs showed very little discrimination between growth phases, maintaining a stable level of expression throughout the observed duration and three candidates 5ʹ UTRs showed a higher expression level in the exponential phase compared to the stationary phase (see Figure 4b).

Not only can these new expression platforms be used as tools for expression during different growth phases in liquid medium, but the expression strength assay may also indicate the temporal expression dynamics of these genes in their native genomic context: As expected, many of the candidate 5ʹ UTRs with higher activity levels in the exponential phase belong to genes expressing proteins involved in central metabolism and proliferation (50S ribosomal protein L25/general stress protein Ctc, RpsL, Ndk [see Figure 4b)].

This is in accordance with previous findings which show that bacterial cells are able to recall distinct global expression patterns based on their stage of growth by the spatio-temporal regulation of chromosomal macrodomains [38]. While replication-induced transient changes in actual copy numbers are a factor directing genomic transcription biases along the oriC/ter axis [39], the regulation of macrodomains occurs for functionally similar genes through direct DNA topology and transcriptional control. While *in trans* expression systems are per definition not affected by positional effects of the 5ʹ UTRs of interest (as they are taken out of their natural, genomic context), they are partially affected by DNA topology [40] (e.g. plasmid supercoiling) and fully affected by transcriptional modulation, under the condition that the entire sequence relevant for regulation is included in the expression system. On the other hand, we see a variety of (often hypothetical proteins) gene functions associated with the candidate 5ʹ UTRs where the expression levels are higher in the stationary compared to the exponential phase. This is a result of the general expression bias in plasmid-based expression systems, which tend to exhibit higher expression levels in the stationary phase. Consequently, a bias towards stationary phase expression can be observed, complicating the interpretation of the native context of these genes and their temporal expression dynamics. This effect is primarily attributed to the enrichment of plasmid copy numbers in the stationary phase relative to the number of cells [10–12]. While saturation of protein density was normalized in our assay by utilizing GFP FI values as a normalization factor for mCherry FI values, a bias introduced due to plasmid copy number enrichment is not. We showed that by harvesting 5ʹ UTRs from the target species genome we were able to create expression systems that behave differently from most synthetic, orthogonal *in trans* expression systems. These expression systems can now be used to further study *Curvibacter* sp. AEP1-3.

The initial library encompassed 500 5ʹ UTR sequences from the *Curvibacter* genome. As 25 of these showed detectable expression levels, the discovery rate is therefore at a minimum of 5%. Many potential promoters may not be active under the artificial laboratory environment and hence show little activity, especially considering that R2A is a complex media that already serves a lot of metabolites and thus requires less *de novo* synthesis of many compounds. To eventually raise the success rate of promoter prediction before manually curating the oligonucleotide library, stretches of sequences around the extracted loci could be used as input sequences for a neural network trained by known promoter sequences such as sequences from the PDD [16]. A similar approach was recently conducted by Seo *et al.* for the cyanobacterial species *Synechocystis* sp. PCC 6803 [41]. The AI-generated prediction could further be used to extract and construct more efficient oligonucleotide sequences. These sequences can be based not only on a continuous DNA-sequence between gene regions but also on specific k-mers of 5ʹ UTR sites. Thus motifs responsible for RNA-polymerase recruitment can be located upstream of the sequences ranging into the next gene sequence, which our approach currently does not cover.

The TF-Motif discovery and enrichment analysis with the XSTREME software (see Method section 2.9) revealed the presence of five enriched transcription factor binding motifs among the 5ʹ UTR sequences. This analysis identified homologous proteins in *Curvibacter* for five of the query transcription factors. Interestingly, some of these homologous transcription factors are located in close proximity to certain 5ʹ UTR sequences. The 5ʹ UTR CPL0025 contains a TF-Motif for the *Pseudomonas aeruginosa* protein LasR, among others. LasR is homologous to CurR2, which is located in two genes downstream of Curl2, the protein associated with CPL0025, suggesting a potential regulatory role for CurR2 based on our dataset. This regulatory function was indeed demonstrated by Pietschke et al. [2]. The presence of homologous proteins and their close proximity to certain 5ʹ UTR sequences suggests that the motifs identified in the tested 5ʹ UTRs may be functionally relevant. These motifs could serve as binding sites for the homologous *Curvibacter* proteins, potentially influencing gene regulation in a manner similar to that observed in the species from which the query proteins originate.

### 4.2 Temporal expression dynamics of highlighted 5ʹ UTRs may correspond with their biological functions

For applications where a stable expression level is essential or accumulation of protein aggregates is a known issue, we recommend the use of the CPL0022 5ʹ UTR, which drives expression of the *dnaK* gene in *Curvibacter* [42]. *dnaK* in *E. coli* is constitutively expressed throughout all of its life cycle and the same seems to account for the *dnaK* equivalent in *Curvibacter* (see Figure 5c). The DnaK protein is a molecular chaperon, a class of enzyme involved in guiding correct folding after translation as well as for already matured proteins. While this maintenance is required constantly, it is generally upregulated when bacteria face external stresses that lead to rapid protein degradation such as heat shocks. Thus, DnaK in *E. coli* is part of the Hsp70 protein group. In *Curvibacter*, the CPL0022 5ʹ UTR also showed a relatively stable expression level throughout all growth phases (see Figure 5c). It would be interesting to see whether this 5ʹ UTR could be utilized as an inducible expression system by applying heat shocks to the cells as a stimulus, effectively acting as an inducible promoter. As *Curvibacter* is studied due to its symbiotic partnership with its host *Hydra vulgaris*, it would be interesting to see whether this 5ʹ UTR also maintains stable activity when growing on the glycocalyx of *Hydra*.

Alternatively, protein aggregation can also be prevented by using a 5ʹ UTR that drives lower expression levels during the stationary phase such as the CPL0095 5ʹ UTR. In its native context, this CPL0095 expresses RpsL, a 12S ribosomal protein of the 30S subunit. This 12S subunit is added late in the biogenesis of the 30S subunit and is essential [43]. Due to the high demand for protein expression during the exponential phase, genes involved in translation are upregulated during that phase, explaining the unusual temporal expression dynamic of this candidate promoter (see Figure 5d). While the expression level of CPL0095 during the exponential growth phase is equal to the strongest sequence CPL0025, it is almost five-fold weaker during the stationary phase in comparison (compare to Figure 5b). Further modification to reduce or enhance the general expression level of this promoter could fine-tune its function for application in continuous cultivation systems such as chemostats. It is likely that other promoters driving gene expression of proteins involved in the assembly of ribosomal subunits or translation are upregulated in a similar fashion and could be a first avenue to find more promoters that behave similarly to CPL0095.

For high levels of expression, we recommend the use of CPL0025 (see Figure 5b) or CPL0017 (see Figure 5a) which has been used in this study as a reference sequence. CPL0025 had the highest level of expression among all tested sequences. Both promoters are well suited for the expression of proteins with very little burden on the host cell metabolism, such as fluorophore proteins for imaging. The gene expressed from CPL0025 functions as an AHL synthase (Curl2), as described by Pietschke et al. [2]. They have shown that the full promoter region (519 bp) of CPL0025 is activated by AHLs produced by *Curvibacter*, as well as by AHLs modified by *Hydra vulgaris*. Here, we show that the shortened promoter region of 76 bp is able to drive a strong expression of our reporter (see Figure 5b). Within the identified transcription factor binding motifs, a TF-Motif similar to a motif discovered for the transcriptional regulator LasR of *Pseudomonas aeruginosa* has been identified [29]. LasR is a LuxR-type regulatory protein and a key component in the quorum sensing system of *Pseudomonas aeruginosa*. LasR binds to AHLs activating the expression of genes involved in various virulence factors and genes important for the adaptation to the environment [44]. However, no LasR TF-Motif can be found within the CPL0025 5’ UTR but a binding motif for the transcriptional regulator CodY from *Lactococcus lactis* and *Streptococcus pyogenes* as well as for CcpA from *Streptococcus pneumoniae*. Those transcriptional regulators are known to regulate the expression of a wide range of genes, e.g. genes responsible for carbohydrate and (p)ppGpp metabolism or virulence factors [35–37]. Regarding the expression of the reporter driven by CPL0025 and the previous finding that the promoter region of *curl2* is activated by AHLs, it is possible that the motif responsible for AHL induction is located within the 76 bp long 5’ UTR of CPL0025. It is unclear whether the high expression level of CPL0025 in the stationary phase are result of transcriptional changes directly related to the growth phase or a result of increasing levels of AHLs in the media or both.

## 5 Outlook

We created a scalable pipeline for the semiautomated discovery of expression systems that can be applied to any bacterial species of interest with an available genome sequence and expression vector. Our workflow was able to find novel expression systems for *Curvibacter* (see Figure 5) which can now be utilized in a variety of applications. It can be extended to specifically search for inducible 5ʹ UTRs, another very relevant manipulation tool for novel model species. First, the entire library could be sorted once under ‘induced’ and once under default conditions and sorted in bulk. After sequencing all 5ʹ UTRs in the expression vector of both populations, the 5ʹ UTRs that appear only in the induced population serve as a list of potential candidates for inducible expression systems. In the same way, this method can be used to find 5ʹ UTRs that are active under any condition of interest, e.g. *Curvibacter* in the presence of the host species *Hydra vulgaris*.

In our curated oligonucleotide library approach, we approximate the rate of RNA polymerase activity by assessing the fluorescence of a reporter protein. In contrast, traditional RNA-seq transcriptome analysis involves counting and comparing mapped read abundances of expressed genes. However, both experimental designs share the common goal of detecting the rate of transcriptional activity under specific environmental conditions. Comparing these strategies, we argue that both methods show different scalability towards distinct scenarios: While for some applications, it is desired to find transcriptional changes of a few genes under a multitude of different circumstances, in other cases the focus may be on global changes in transcription levels for only a handful of environmental circumstances. Traditional RNAseq workflows allow for a global analysis of a transcriptome but the amount of samples increases linearly with the amount of observed conditions. To maintain an adequate sequencing depth for each individual sample, this leads to scaling sequencing costs based on the amount of samples. Vice versa, our oligonucleotide-based screening workflow can be very well adapted to screen many conditions in a single workflow, as cultivation capacities and the scalability of flow cytometry based sorting are the only rate-limiting factors. Another advantage is that once positive candidates are sorted and sequenced, the vectors for further studies are already available.

## Supplementary Material

ysaf001_Supp

## Data Availability

All Code used in this manuscript as well as raw data from bulk fluorescence measurements is available under (https://github.com/Kanomble/curvibacter_promotor_studies). A GenBank file of the entry vector used to clone the promoter library is available in the GitHub data repository, including highlights for restriction sites.
