# Supplementary material for: Oligonucleotide library assisted sequence mining reveals promoter sequences with distinct temporal expression dynamics for applications in Curvibacter sp. AEP1-3
Source: Synth Biol (Oxf). 2025 May 21;10(1):ysaf001. doi: 10.1093/synbio/ysaf001 (PMC12094071; doi:10.1093/synbio/ysaf001)
Supplement: ysaf001_Supp [file ysaf001_supp.zip › suppl_data/Supplementary_Material.pdf]

Supplements: Oligonucleotide Library Assisted Sequence Mining Reveals Promoter Sequences With Distinct Temporal Expression Dynamics For Applications In *Curvibacter* sp. AEP1-3

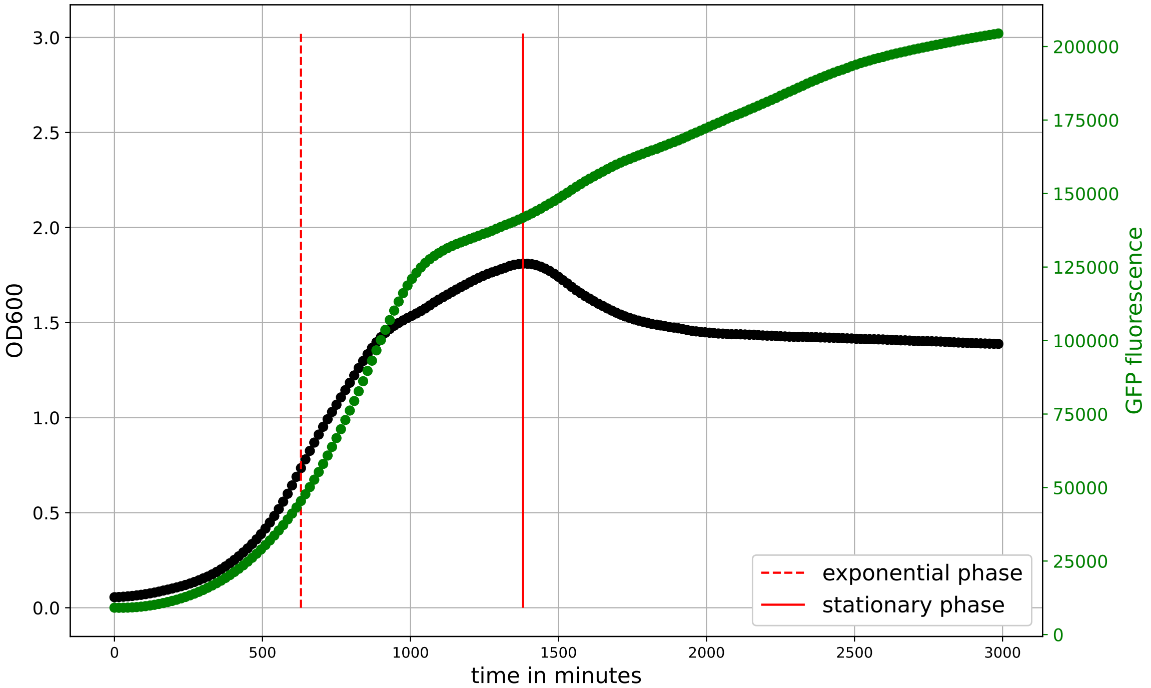

Supplementary Figure S1: Comparison of change in biomass and GFP expression in *Curvibacter* sp. AEP1-3 glmS::GFP strain carrying CPL0022 reporter plasmid.

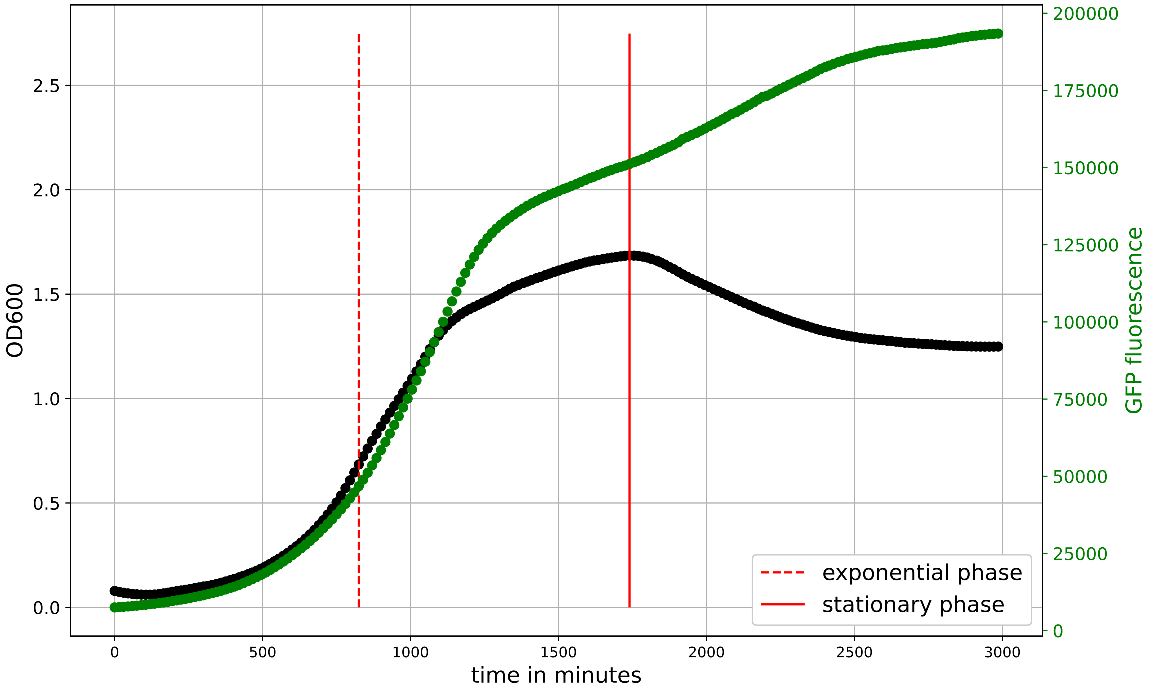

Supplementary Figure S2: Comparison of change in biomass and GFP expression in *Curvibacter* sp. AEP1-3 glmS::GFP strain carrying CPL0017 reporter plasmid.

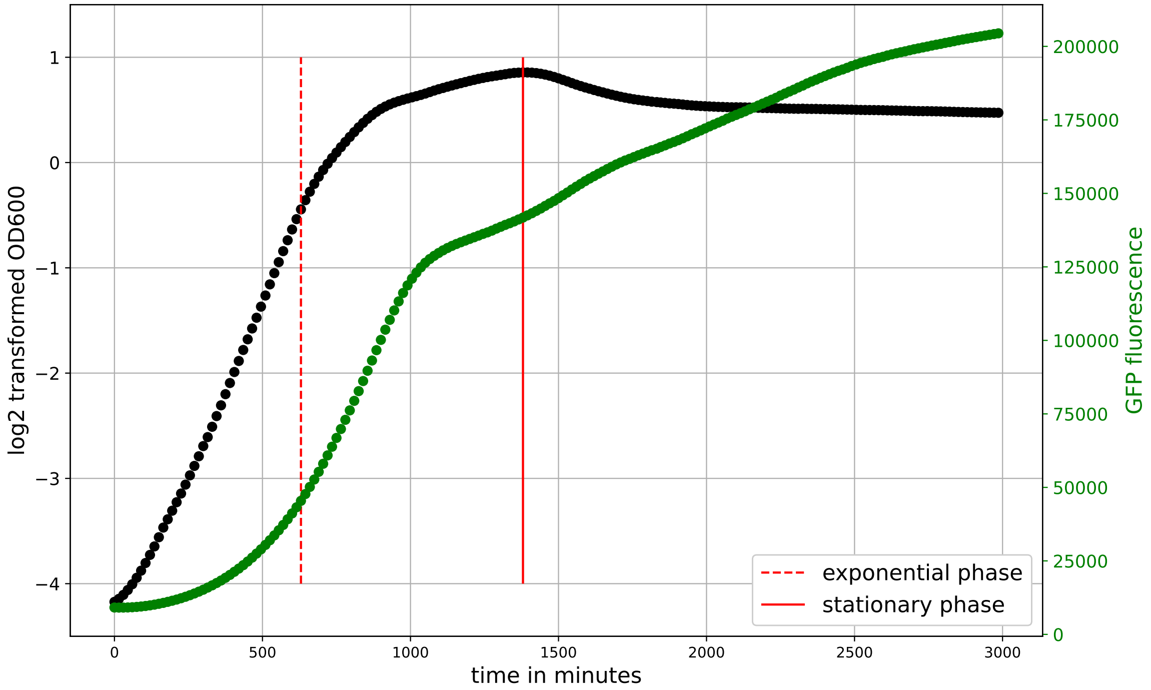

Supplementary Figure S3: Comparison of change in biomass and GFP expression in *Curvibacter* sp. AEP1-3 glmS::GFP strain carrying CPL0017 reporter plasmid, on log scale.

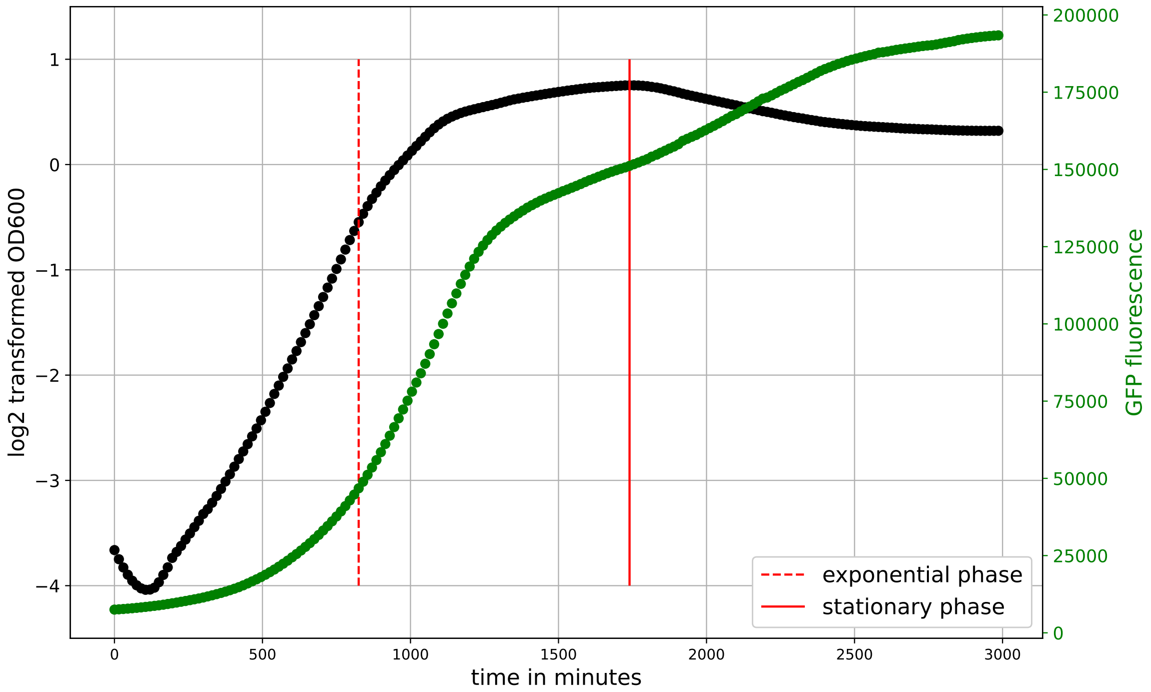

Supplementary Figure S4: Comparison of change in biomass and GFP expression in *Curvibacter* sp. AEP1-3 glmS::GFP strain carrying CPL0022 reporter plasmid, on log scale

Supplementary Table S1: Extended list of detected motifs in the sorted candidates after cell sorting by flow cytometry.

	CPL	motif_ID	site_Start	site_End	site_Sequence	similarMotifs
0	CPL0001	3-TTATGAAAAA	11	20	TTATGCAAAA	CodY: S.pyogenes; CodY: L.lactis; CcpA: S.pneumoniae
1	CPL0001	2-CCGAAAAGYCT	46	56	CCGATAACTCT	CodY: B. anthracis
2	CPL0001	4-CTACAGKAA	72	80	CTACGGTAA	LexA: V.parahaemolyticus
3	CPL0002	3-TTATGAAAAA	5	14	TTATAAGAAA	CodY: S.pyogenes; CodY: L.lactis; CcpA: S.pneumoniae
4	CPL0003	3-TTATGAAAAA	53	62	CTATCAGAAC	CodY: S.pyogenes; CodY: L.lactis; CcpA: S.pneumoniae
5	CPL0004	3-TTATGAAAAA	59	68	TTATGAAGAG	CodY: S.pyogenes; CodY: L.lactis; CcpA: S.pneumoniae
6	CPL0004	4-CTACAGKAA	4	12	CTACAGTGG	LexA: V.parahaemolyticus
7	CPL0005	2-CCGAAAAGYCT	29	39	CCCAAAGGCCT	CodY: B. anthracis
8	CPL0006	2-CCGAAAAGYCT	12	22	CCCAATAGCCT	CodY: B. anthracis
9	CPL0006	3-TTATGAAAAA	73	82	TTATGAAAAA	CodY: S.pyogenes; CodY: L.lactis; CcpA: S.pneumoniae
10	CPL0006	4-CTACAGKAA	122	130	CCACAGGTA	LexA: V.parahaemolyticus
11	CPL0006	2-CCGAAAAGYCT	31	41	CCCAAAACCCT	CodY: B. anthracis
12	CPL0008	1-CGCGASCAGY	2	11	GGCGACCACT	AmrZ,LasR: P. aeruginosa; BldD: S. coelicolor
13	CPL0008	5-CAAGYSCCYRMMHB	43	56	CAAGTGCCTACACT	ExpR: S. meliloti; DosR: M.tuberculosis
14	CPL0008	3-TTATGAAAAA	65	74	TTAAGAAAAC	CodY: S.pyogenes; CodY: L.lactis; CcpA: S.pneumoniae
15	CPL0008	4-CTACAGKAA	18	26	CTACAGGAA	LexA: V.parahaemolyticus
16	CPL0012	1-CGCGASCAGY	46	55	GACGACCTGT	AmrZ,LasR: P. aeruginosa; BldD: S. coelicolor
17	CPL0012	3-TTATGAAAAA	14	23	CTATGCAAAA	CodY: S.pyogenes; CodY: L.lactis; CcpA: S.pneumoniae
18	CPL0012	2-CCGAAAAGYCT	82	92	ACGACAACCAT	CodY: B. anthracis
19	CPL0021	1-CGCGASCAGY	4	13	CGCCACCGGT	AmrZ,LasR: P. aeruginosa; BldD: S. coelicolor
20	CPL0022	1-CGCGASCAGY	12	21	CACAACCAGC	AmrZ,LasR: P. aeruginosa; BldD: S. coelicolor
21	CPL0022	2-CCGAAAAGYCT	105	115	CCTAAAACTCT	CodY: B. anthracis
22	CPL0022	5-CAAGYSCCYRMMHB	48	61	CAAGTGCCCACAAG	ExpR: S. meliloti; DosR: M.tuberculosis
23	CPL0025	3-TTATGAAAAA	20	29	TTACAAGAAA	CodY: S.pyogenes; CodY: L.lactis; CcpA: S.pneumoniae
24	CPL0055	1-CGCGASCAGY	41	50	CGCGAGTAGC	AmrZ,LasR: P. aeruginosa; BldD: S. coelicolor
25	CPL0055	3-TTATGAAAAA	2	11	CTATCAAAAA	CodY: S.pyogenes; CodY: L.lactis; CcpA: S.pneumoniae
26	CPL0065	5-CAAGYSCCYRMMHB	10	23	CAAGCGCCTACCCC	ExpR: S. meliloti; DosR: M.tuberculosis

27	CPL0065	3-TTATGAAAAA	74	83	TTATGAAACT	CodY: S.pyogenes; CodY: L.lactis; CcpA: S.pneumoniae
28	CPL0066	2-CCGAAAAGYCT	3	13	CCGAACAGCAA	CodY: B. anthracis
29	CPL0066	1-CGCGASCAGY	32	41	TGCGATCTGT	AmrZ,LasR: P. aeruginosa; BldD: S. coelicolor
30	CPL0067	4-CTACAGKAA	46	54	CTACCGGAG	LexA: V.parahaemolyticus
31	CPL0067	5-CAAGYSCCYRMMHB	10	23	TAAGCCCCTGCTCC	ExpR: S. meliloti; DosR: M.tuberculosis
32	CPL0068	2-CCGAAAAGYCT	29	39	ACGATAAGCCC	CodY: B. anthracis
33	CPL0068	4-CTACAGKAA	62	70	CAACCGGAA	LexA: V.parahaemolyticus
34	CPL0069	3-TTATGAAAAA	10	19	TTACAAAAAC	CodY: S.pyogenes; CodY: L.lactis; CcpA: S.pneumoniae
35	CPL0070	5-CAAGYSCCYRMMHB	30	43	GAAGCGCATAAAAT	ExpR: S. meliloti; DosR: M.tuberculosis
36	CPL0071	4-CTACAGKAA	13	21	CCACTGTAA	LexA: V.parahaemolyticus
37	CPL0071	1-CGCGASCAGY	43	52	CACGACGTGT	AmrZ,LasR: P. aeruginosa; BldD: S. coelicolor
38	CPL0072	1-CGCGASCAGY	23	32	CGCAGGCAGT	AmrZ,LasR: P. aeruginosa; BldD: S. coelicolor
39	CPL0073	3-TTATGAAAAA	76	85	TTATGTAGAA	CodY: S.pyogenes; CodY: L.lactis; CcpA: S.pneumoniae
40	CPL0073	1-CGCGASCAGY	38	47	CGCGAGCAGC	AmrZ,LasR: P. aeruginosa; BldD: S. coelicolor
41	CPL0073	3-TTATGAAAAA	24	33	CTATGAAAAC	CodY: S.pyogenes; CodY: L.lactis; CcpA: S.pneumoniae
42	CPL0074	5-CAAGYSCCYRMMHB	15	28	CTAGCGCCCGTCTT	ExpR: S. meliloti; DosR: M.tuberculosis
43	CPL0074	2-CCGAAAAGYCT	1	11	CCGAAAAGTCT	CodY: B. anthracis
44	CPL0074	1-CGCGASCAGY	34	43	CGCGAGCAGC	AmrZ,LasR: P. aeruginosa; BldD: S. coelicolor
45	CPL0074	3-TTATGAAAAA	48	57	CTATAAAAAT	CodY: S.pyogenes; CodY: L.lactis; CcpA: S.pneumoniae
46	CPL0075	5-CAAGYSCCYRMMHB	45	58	GAAGCTCCTAAAAG	ExpR: S. meliloti; DosR: M.tuberculosis
47	CPL0077	1-CGCGASCAGY	3	12	CGCAAGCAGC	AmrZ,LasR: P. aeruginosa; BldD: S. coelicolor
48	CPL0080	5-CAAGYSCCYRMMHB	1	14	CAAGTCCGTCAACG	ExpR: S. meliloti; DosR: M.tuberculosis
49	CPL0081	5-CAAGYSCCYRMMHB	35	48	CAAGCCCCCGCCTC	ExpR: S. meliloti; DosR: M.tuberculosis
50	CPL0081	2-CCGAAAAGYCT	73	83	CCGAAGACCCT	CodY: B. anthracis
51	CPL0083	1-CGCGASCAGY	66	75	CGCGACCAGT	AmrZ,LasR: P. aeruginosa; BldD: S. coelicolor
52	CPL0083	4-CTACAGKAA	50	58	CGACAGTAA	LexA: V.parahaemolyticus
53	CPL0095	2-CCGAAAAGYCT	47	57	CCGAAAAGCCT	CodY: B. anthracis
54	CPL0095	4-CTACAGKAA	37	45	CTACATTAG	LexA: V.parahaemolyticus
55	CPL0095	1-CGCGASCAGY	5	14	CGCAAGCAGT	AmrZ,LasR: P. aeruginosa; BldD: S. coelicolor
56	CPL0095	3-TTATGAAAAA	77	86	TTACGAAAAC	CodY: S.pyogenes; CodY: L.lactis; CcpA: S.pneumoniae
57	CPL0106	3-TTATGAAAAA	83	92	TTATGAAAAA	CodY: S.pyogenes; CodY: L.lactis; CcpA: S.pneumoniae

58	CPL0106	1-CGCGASCAGY	8	17	CGCGAGCAGC	AmrZ,LasR: P. aeruginosa; BldD: S. coelicolor
59	CPL0106	2-CCGAAAAGYCT	48	58	CCTAAAAGCCA	CodY: B. anthracis
60	CPL0109	2-CCGAAAAGYCT	51	61	CCCAAAAGTAT	CodY: B. anthracis
61	CPL0112	4-CTACAGKAA	18	26	GTACAGGAA	LexA: V.parahaemolyticus
